# Supplementary material for: Causal Relationship between Aging and Anorexia Nervosa: A White-Matter-Microstructure-Mediated Mendelian Randomization Analysis
Source: Biomedicines. 2024 Aug 16;12(8):1874. doi: 10.3390/biomedicines12081874 (PMC11351342; doi:10.3390/biomedicines12081874)
Supplement: Supplementary file 1 [file biomedicines-12-01874-s001.zip › Supplementary Figure/Figure S4/Singe SNP plot of MR analysis for TL to ubm-b-1773.pdf]

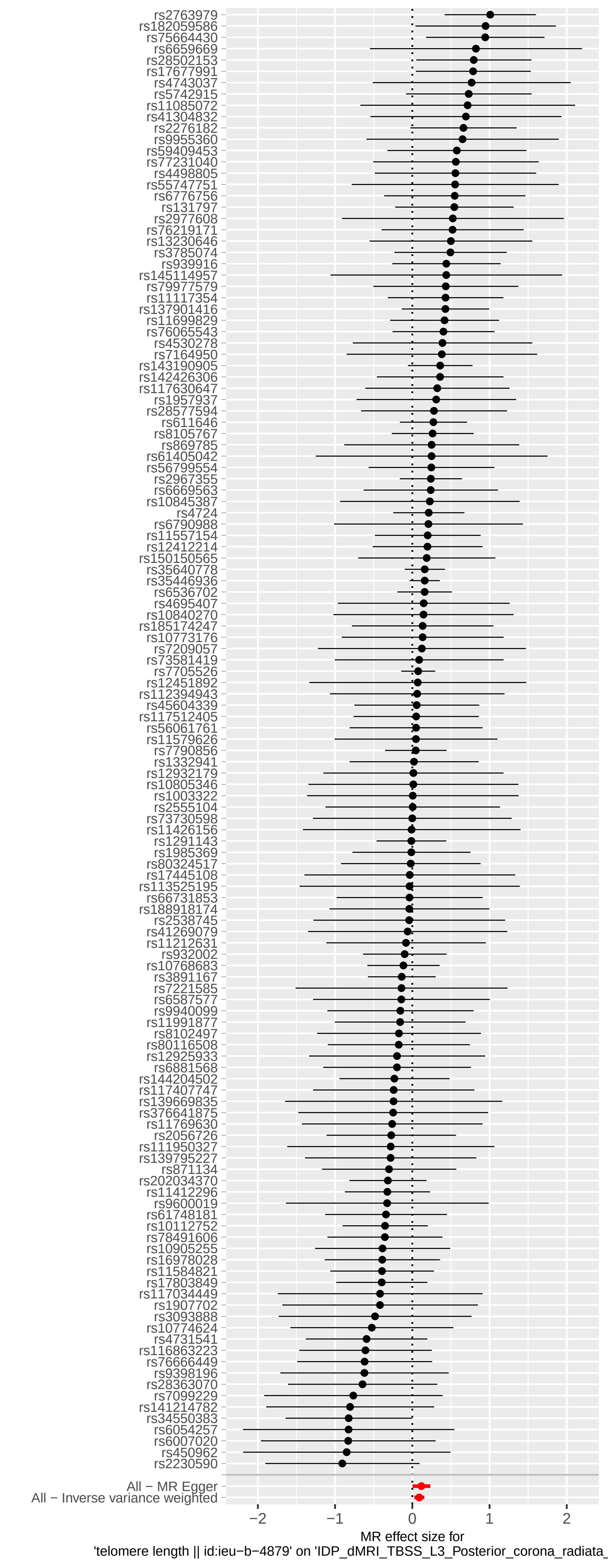
rs2763979
rs182059586
rs75664430
rs6659669
rs28502153
rs17677991
rs4743037
rs5742915
rs11085072
rs41304832
rs2276182
rs9955360
rs59409453
rs77231040
rs4498805
rs55747751
rs6776756
rs131797
rs2977608
rs76219171
rs13230646
rs3785074
rs939916
rs145114957
rs79977579
rs11117354
rs137901416
rs11699829
rs76065543
rs4530278
rs7164950
rs143190905
rs142426306
rs117630647
rs1957937
rs28577594
rs611646
rs8105767
rs869785
rs61405042
rs56799554
rs2967355
rs6669563
rs10845387
rs4724
rs6790988
rs11557154
rs12412214
rs150150565
rs35640778
rs35446936
rs6536702
rs4695407
rs10840270
rs185174247
rs10773176
rs7209057
rs73581419
rs7705526
rs12451892
rs112394943
rs45604339
rs117512405
rs56061761
rs11579626
rs7790856
rs1332941
rs12932179
rs10805346
rs1003322
rs2555104
rs73730598
rs11426156
rs1291143
rs1985369
rs80324517
rs17445108
rs113525195
rs66731853
rs188918174
rs2538745
rs41269079
rs11212631
rs932002
rs10768683
rs3891167
rs7221585
rs6587577
rs9940099
rs11991877
rs8102497
rs80116508
rs12925933
rs6881568
rs144204502
rs117407747
rs139669835
rs376641875
rs11769630
rs2056726
rs111950327
rs139795227
rs871134
rs202034370
rs11412296
rs9600019
rs61748181
rs10112752
rs78491606
rs10905255
rs16978028
rs11584821
rs17803849
rs117034449
rs1907702
rs3093888
rs10774624
rs4731541
rs116863223
rs76666449
rs9398196
rs28363070
rs7099229
rs141214782
rs34550383
rs6054257
rs6007020
rs450962
rs2230590
All − MR Egger
All − Inverse variance weighted
−2
−1
0
1
2
MR effect size for
'telomere length || id:ieu−b−4879' on 'IDP_dMRI_TBSS_L3_Posterior_corona_radiata_
